# Peritoneal Carcinomatosis From Malignant Melanoma After Wide Local Excision of a T1a Lesion: A Case Report

**DOI:** 10.7759/cureus.35730

**Published:** 2023-03-03

**Authors:** Matthew Hager, John Miller, Adam Wegener, James Yon

**Affiliations:** 1 General Surgery, Novant Health New Hanover Regional Medical Center, Wilmington, USA

**Keywords:** cutaneous malignant melanoma, lactate dehydrogenase, metastatic melanoma, skin cancer, peritoneal carcinomatosis, peritoneal metastasis, melanoma

## Abstract

Cutaneous melanoma is an aggressive skin cancer with a high propensity for distant metastasis. Peritoneal metastasis from cutaneous malignant melanoma is extremely rare and associated with a low five-year overall survival rate. This report reviews a case of a 78-year-old man with peritoneal carcinomatosis after a wide local excision of a T1a invasive malignant melanoma of the scalp 10 months prior. Although rare, patients with a recent history of melanoma who present with obscure or odd symptoms should be worked up for metastatic disease by their surgeon or medical oncologist. Carcinomatosis can develop, and early treatment with immunotherapy has been shown to improve five-year overall survival and progression-free survival in patients with advanced melanoma.

## Introduction

Cutaneous malignant melanoma is an aggressive skin cancer with a propensity for regional and distant metastasis. Melanoma is the fifth most common malignancy, and it is estimated that 7,180 deaths from melanoma will occur in the US this year. Common risk factors associated with the diagnosis include Caucasian males, typically of older age. From 1975 to 2018, the incidence of melanoma in the US increased by greater than 320%, from 7.9/100,000 in 1975 to 25.3/100,000 in 2018. The prognosis for patients diagnosed with stage I/II disease is excellent, with a five-year relative survival rate of 99.4%. And while patients with a nodal or distant disease have a worse prognosis, the advent of immunotherapy has made significant improvements in overall survival [1].

Patients with a history of malignant melanoma require routine surveillance with a focused physical exam performed by either a dermatologist or a surgeon at regularly scheduled intervals. These patients are not only at increased risk for recurrence and distant disease, but they are also 9x more likely to develop a subsequent melanoma compared with the general population [2]. In that same light, clinicians must maintain a high index of suspicion for metastatic disease in this patient population due to its propensity for distant spread remote from the index skin lesion.

Cutaneous melanoma spreads through either a direct extension or invasion of neighboring cells, lymphatic channels, or hematogenously [3]. Peritoneal carcinomatosis is a rare presentation of melanoma and is caused by hematogenous spread. A population study of patients with peritoneal metastasis found that 0.5% were caused by melanoma [3]. In this report, the authors present details of a patient with peritoneal carcinomatosis from a cutaneous melanoma, who presented with abdominal pain and ascites several months after an R0 resection of a scalp lesion.

## Case presentation

RS is a 78-year-old male who presented to the surgical oncology clinic in early 2020 with newly diagnosed melanoma of the right superior parietal scalp. The patient underwent a shave biopsy of a black macular scalp lesion in the dermatologist’s office. The biopsy revealed melanoma in situ and invasive malignant melanoma with a Clark's Level II, maximal tumor thickness of 0.74 mm, without ulceration or evidence of microsatellitetosis, with margins narrowly clear. A physical exam was performed, including a full body skin exam and nodal basin evaluation. No additional skin lesions were identified, and there was no evidence of lymphadenopathy on palpation.

At that time, the patient reported a history of sun exposure but denied previous sunburns or the use of tanning beds. The patient’s past medical history included stage I renal cell carcinoma status post left nephrectomy in 2012; early-stage adenocarcinoma of the lung treated with left lobectomy in 2015 without chemotherapy or radiation; squamous cell carcinoma of the nose and basal cell carcinoma of the right shoulder status post excision; hypertension; hyperlipidemia; and degenerative disc disease. He reported a history of tobacco use with a 14-pack/year smoking history having quit in 1975. He also endorsed ethyl alcohol (EtOH) consumption of two to three drinks/day for the past 20+ years.

On February 18, 2020, he underwent a wide local excision of the scalp lesion with 1 cm margins. Postoperative pathology revealed no evidence of residual melanoma.

In September 2020, the patient had a computed tomography (CT) of his chest, abdomen, and pelvis for continued monitoring of multiple pulmonary nodules. A subtle hypo-enhancing right hepatic lobe lesion and left hepatic lobe density with parenchymal calcifications were noted, in addition to his known pulmonary nodules. 

**Figure 1:**
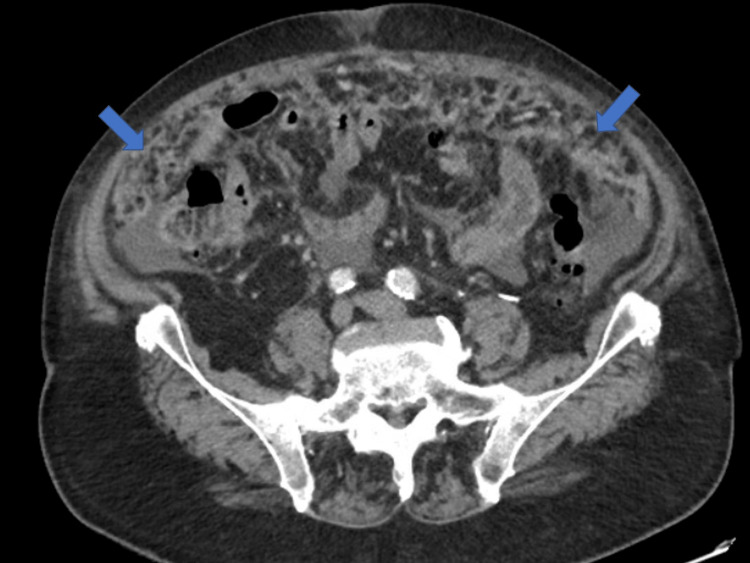
CT abdomen and pelvis with omental caking on December 22, 2020

In December 2020, he presented to an outside hospital with a four-week history of progressive abdominal distention, abdominal pain, bilateral lower extremity edema, and anorexia. An abdominal CT scan revealed moderate abdominal ascites, diffuse omental and peritoneal carcinomatosis, and lytic bony lesions in the spine and pelvic bones, causing concern for metastasis (Figure 1). During that hospitalization, he underwent ultrasound-guided paracentesis with 2.4 liters of cloudy yellow ascitic fluid removed. Cytology of the fluid showed no malignant cells. No biopsy was performed during his hospitalization. The patient’s symptoms improved, his electrolyte abnormalities, including hyponatremia, hypomagnesemia, and hypokalemia were corrected, and he was subsequently discharged from the hospital on Aldactone and Furosemide.

**Figure 2:**
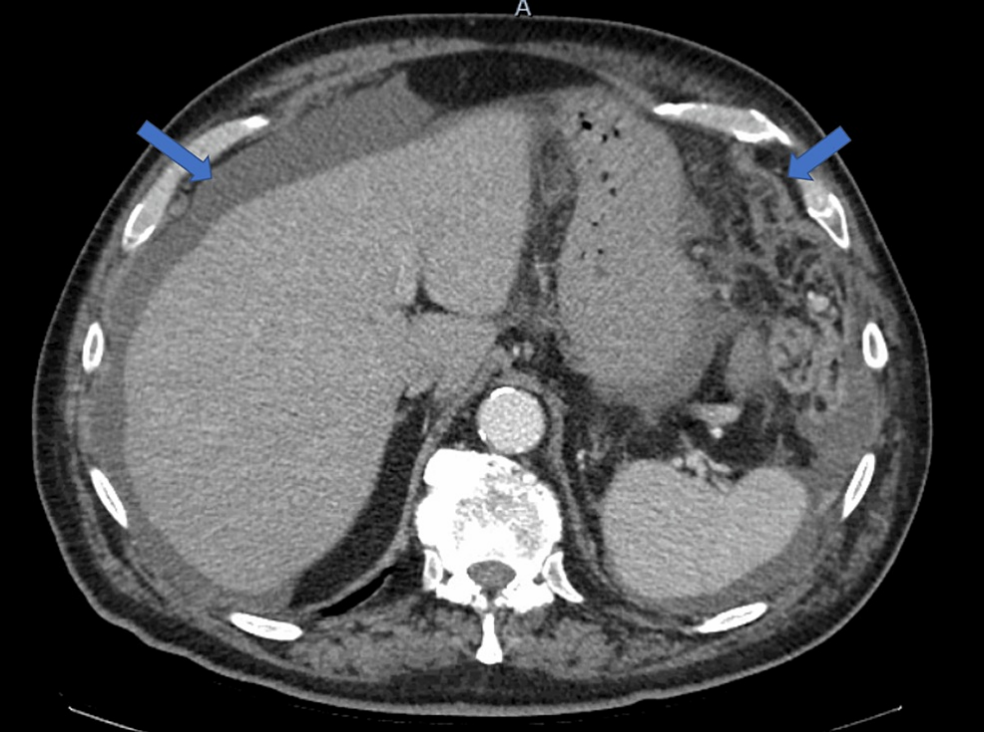
Abdominal ascites and nodularity of the omentum suggestive of peritoneal carcinomatosis seen on chest CT on December 28, 2020

**Figure 3:**
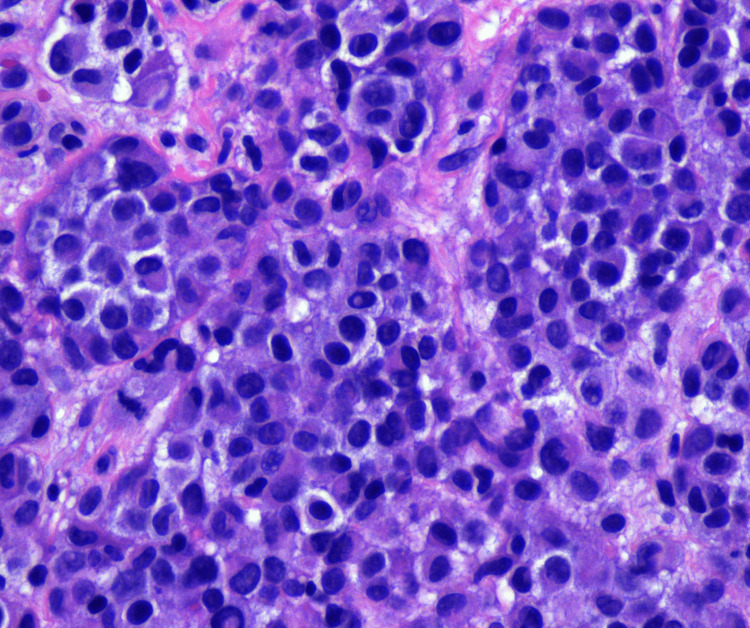
H&E stain of peritoneal biopsy

**Figure 4:**
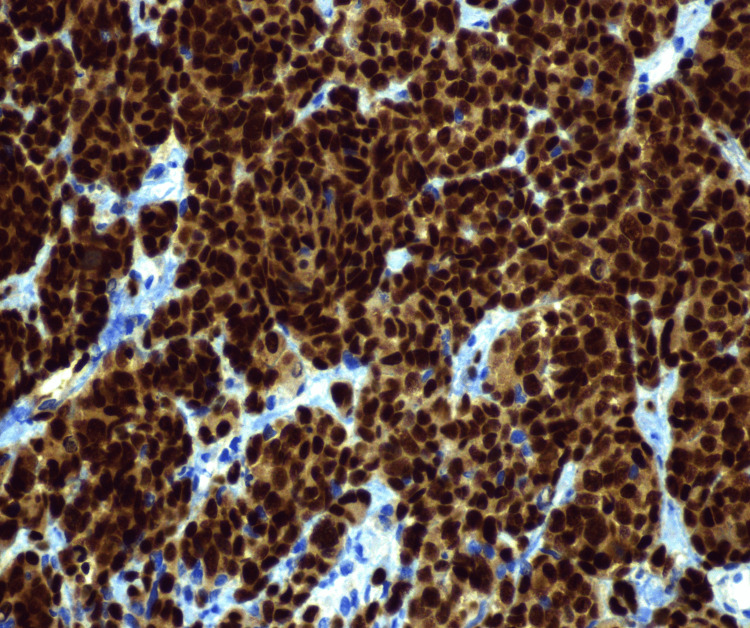
SOX-10 stain of peritoneal biopsy

The patient underwent repeat imaging four days after his discharge. This revealed increasing pulmonary nodule size and number, previously seen hepatic lesions, abdominal ascites, new small lucent bone lesions, and a nodular lesion in the omentum, which was concerning for peritoneal carcinomatosis (Figure 2). The patient’s carcinoembryonic antigen (CEA) and cancer antigen (CA) 19-9 were 1.77 ng/mL and 13.52 U/mL, respectively. Lactate dehydrogenase (LDH) measured 361 U/L, which was elevated from 176 U/L one year prior. After a follow-up with medical oncology staff, the patient was referred to the surgical oncology clinic for port placement and diagnostic laparoscopy with peritoneal biopsy for a definitive diagnosis. On January 5, 2021, the patient underwent a diagnostic laparoscopy that revealed extensive peritoneal carcinomatosis with lesions on the liver, bladder, omentum, peritoneum, and bowel. Pathology from an omental biopsy was consistent with malignant melanoma on hematoxylin and eosin (H&E) stain (Figure 3). Immunohistochemistry and special stains revealed S-100 positive, SOX-10 positive (Figure 4), Melan A/MART 1 positive, PanKeratin negative, CAM 5.2 negative, BRAF V600k mutation-positive, C-Kit negative, MS-stable, and HMB45 focally positive.

Postoperatively, the patient was scheduled to undergo a positron emission tomography (PET)/CT scan and magnetic resonance imaging (MRI) of the brain to complete his staging. Medical oncology had recommended the initiation of pembrolizumab. However, his nutritional status continued to worsen, and the patient ultimately expired prior to the initiation of immunotherapy or obtaining further imaging studies.

## Discussion

We describe an exceedingly rare case of a 78-year-old male who was diagnosed with an early-stage IA melanoma and underwent wide excision with 1 cm margins, and within 10 months, was found to have a widely metastatic disease with carcinomatosis. The biology of his tumor was obviously aggressive in nature. His presentation raises the question as to whether there is an opportunity for an earlier diagnosis of the progression of his disease. According to National Comprehensive Cancer Network (NCCN) guidelines, patients with stage IA disease are typically recommended to undergo surveillance with a focused history and physical every six to 12 months, with imaging warranted only to investigate specific symptoms [4]. While this patient received the standard of care, his presentation highlights the importance of asking all melanoma patients pertinent questions to elucidate the presence of metastatic disease, which might initiate more imaging at the time of his initial presentation or at his six-month follow-up. As the most common sites of metastasis are the lungs, liver, lymph nodes, brain, and bones [4], the history and physical exam should focus on the presence or absence of symptoms related to these systems.

Prior studies that have focused on melanoma of the head and neck have demonstrated a worse overall prognosis [5-8]. And those melanomas that are confined to the scalp, even more so. In fact, one study reported on multivariate analysis that tumor sites on the scalp had more than three-fold greater mortality than tumors on the face [6]. Given that our patient presented with a T1a lesion, current NCCN guidelines do not recommend sentinel lymph node biopsy (SLNB) as the risk of nodal metastasis is less than 5% [4]. However, in the setting of a patient who presents with scalp melanoma, with a history of multiple prior malignancies, perhaps it would have been prudent to discuss the performance of an SLNB at the time of the index-wide excision. It is not to say that this would have necessarily altered the patient’s outcome, as the majority of peritoneal carcinomatosis occurs from hematogenous spread [3] but instead emphasizes the fact that clinicians should have a higher index of suspicion for aggressive disease when the primary lesion is localized to the scalp. Moreover, while the guidelines are put in place to help direct the treatment that patients should receive, each patient should be approached as an individual, considering all of their pertinent histories.

Elevated serum lactate dehydrogenase (LDH) is one biomarker that has been found to be a strong independent prognostic factor in metastatic melanoma. Elevated LDH has been shown to correlate with decreased survival in patients with advanced metastatic melanoma. It has also recently been used, over the past few years, as a marker of response to therapy [9]. One drawback of LDH is that it is only elevated in stage IV disease and has not been seen to have a role in the early spread of the disease. This is evidenced by normal LDH levels seen in patients with distant subcutaneous spread of melanoma and even normal values noted in patients with pulmonary metastasis [10].

One study found that in 50% of patients with peritoneal carcinomatosis, clinical signs of ascites and bowel obstruction were present. The addition of the presence of neoplastic cells within the ascitic fluid helps confirm the diagnosis [11]. In this patient’s case, there were clinical findings of ascites and abdominal pain, but there were no neoplastic cells seen within the ascitic fluid on paracentesis. This case supports the variability found in clinical presentations of malignant melanoma with peritoneal metastasis.

According to Flanagan et al., the mean global survival of patients with stage IV melanoma diagnosed with peritoneal implants was 1.8 months, compared to 12.3 months in patients with stage IV melanoma without peritoneal involvement [3]. Surgery and radiation therapy have limited roles in the treatment of metastatic melanoma, as the mainstay of treatment remains immunotherapy [4].

According to the NCCN, first-line therapy for metastatic or unresectable malignant melanoma is monotherapy with anti-PD1 agents (nivolumab, pembrolizumab) or a combination of targeted therapies for BRAF V600 (dabrafenib, trametinib, and vemurafenib) [4]. In a five-year follow-up study of patients with stage IV melanoma who received pembrolizumab monotherapy, overall survival at five years was 34% in all patients and 41% in treatment-naïve patients. Progression-free survival (PFS) in these patients was lower with a five-year PFS of 21% in all patients and 29% in treatment-naive patients [12]. Shortly thereafter, in 2019, Larkin et al. found an overall survival of 52% at five years in patients with stage IV melanoma who received nivolumab plus ipilimumab therapy compared to 26% in the ipilimumab alone group [13]. These newer immunotherapeutic agents have shifted the paradigm for patients with advanced-stage melanoma, greatly improving overall survival and progression-free survival.

## Conclusions

Although invasive malignant melanoma with peritoneal carcinomatosis is extremely rare, patients with a melanoma history who present with obscure or odd symptoms should be considered to have metastatic disease until proven otherwise. These patients should be referred to their surgeon or medical oncologist and likely warrant full staging imaging (PET/CT, brain MRI), as carcinomatosis can develop and data on ideal treatment are limited. However, with the advent of immunotherapy, and the benefit it has shown in stage III and IV disease, these patients should attempt to initiate immunotherapy as soon as possible.
